# Stringent Response in Mycobacteria: From Biology to Therapeutic Potential

**DOI:** 10.3390/pathogens10111417

**Published:** 2021-11-01

**Authors:** Kuldeepkumar Ramnaresh Gupta, Gunjan Arora, Abid Mattoo, Andaleeb Sajid

**Affiliations:** 1 Department of Microbial Pathogenesis, Yale University School of Medicine, New Haven, CT 06520, USA; kuldeepkumar.gupta@yale.edu; 2 Section of Infectious Diseases, Department of Internal Medicine, Yale University School of Medicine, New Haven, CT 06520, USA; gunjan.arora@yale.edu; 3 Pharmaceutical Development, Ultragenyx Gene Therapy, Woburn, MA 01801, USA; abidmattoo@gmail.com

**Keywords:** *Mycobacterium*, alarmones, (pp)pGpp. Rel, RelZ, stress response, drug resistance, biofilm, virulence, stringent response

## Abstract

*Mycobacterium tuberculosis* is a human pathogen that can thrive inside the host immune cells for several years and cause tuberculosis. This is due to the propensity of *M. tuberculosis* to synthesize a sturdy cell wall, shift metabolism and growth, secrete virulence factors to manipulate host immunity, and exhibit stringent response. These attributes help *M. tuberculosis* to manage the host response, and successfully establish and maintain an infection even under nutrient-deprived stress conditions for years. In this review, we will discuss the importance of mycobacterial stringent response under different stress conditions. The stringent response is mediated through small signaling molecules called alarmones “(pp)pGpp”. The synthesis and degradation of these alarmones in mycobacteria are mediated by Rel protein, which is both (p)ppGpp synthetase and hydrolase. Rel is important for all central dogma processes—DNA replication, transcription, and translation—in addition to regulating virulence, drug resistance, and biofilm formation. Rel also plays an important role in the latent infection of *M. tuberculosis*. Here, we have discussed the literature on alarmones and Rel proteins in mycobacteria and highlight that (p)ppGpp-analogs and Rel inhibitors could be designed and used as antimycobacterial compounds against *M. tuberculosis* and non-tuberculous mycobacterial infections.

## 1. Introduction

**Figure 1:**
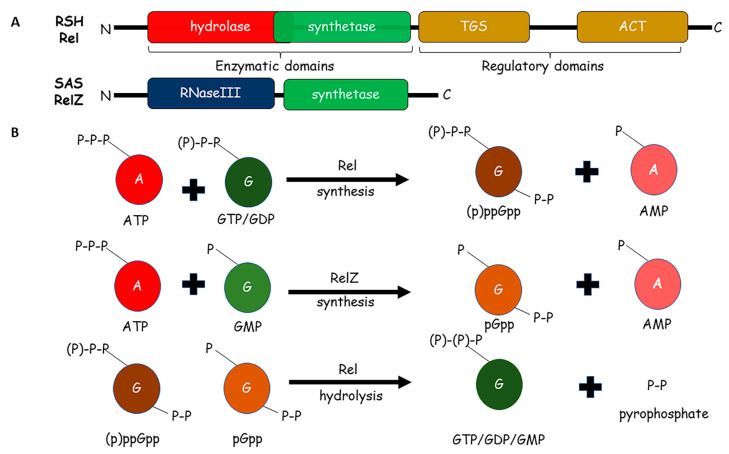
Synthesis and degradation of alarmones: (**A**) Domain architecture of RSH-Rel and SAS- RelZ proteins involved in synthesis and hydrolysis of alarmones (pp)pGpp. Rel is composed of two enzymatic (hydrolase and synthetase) and two regulatory (TGS and ACT) domains. RelZ has N-terminal RNaseIII domain, which is followed by the synthetase domain. (**B**) Metabolism of alarmones by Rel and RelZ enzymes. The synthesis steps utilize ATP and guanine nucleotides as precursors. The hydrolysis results in the formation of the same guanine nucleotide in addition to di-phosphate or pyrophosphate.

Bacteria encounter constantly changing environments that may threaten their survival and existence; hence, it is particularly important to study their survival strategies in different model systems [1,2,3]. These strategies include several sensory mechanisms and signaling pathways that are required to overcome such threats [4,5,6,7,8,9]. These mechanisms help bacteria to sense the environmental cues and generate an appropriate adaptive response. The adaptive response is usually multilayered and may affect some or all aspects of metabolism, replication, transcription, translation, and post-translational modifications in bacteria [5,10,11,12,13]. Hence, a prompt adaptation to such abrupt changes becomes a necessity for bacterial survival. The stringent response is one such evolutionarily conserved mechanism, through which bacteria can thrive in hostile conditions [13]. It is mediated through small molecules called alarmones, which include tetraphosphate guanosine and pentaphosphate guanosine, collectively referred to as (p)ppGpp [14] (Figure 1). Stringent response affects all the central dogma processes—replication, transcription, and translation [15]. It helps bacteria survive the stress conditions by regulating important processes, such as biofilm formation, antibiotic resistance, persistence, and virulence in bacterial pathogens [15,16,17].

The mediators of stringent response, (p)ppGpp, were first identified in 1969 when the nucleotide extracts of the amino-acid-starved cultures of *Escherichia coli* K-12 were resolved by thin-layer chromatography [18]. While native culture extracts showed two spots corresponding to ppGpp and pppGpp, these so-called “magic spots” were absent in the extracts from a mutant that had unregulated rRNA synthesis. Since this particular strain, a methionine auxotroph, then known as “58-161” mutant, exhibited the unregulated or relaxed synthesis of rRNA even during the amino acid starvation, the associated locus was called “*relA*” [19]. Classically, the stringent response has been associated with the synthesis of alarmones to stop rRNA production during amino acid starvation in *E.* *coli* [20,21,22,23]. However, subsequent research has shown that several bacterial phyla, including actinobacteria, produce alarmones, (p)ppGpp, upon amino acid starvation [13,16,24,25,26,27,28]. Additionally, in recent years, a third alarmone, pGpp, has been discovered in several bacteria, which has further expanded the repertoire of stringent response [29,30,31,32,33,34]. Thus, the three alarmones—pGpp, ppGpp, and pppGpp—are now collectively referred to as (pp)pGpp and are mediators of stringent response in bacteria.

Mycobacteria comprise various obligate human pathogens such as *M. tuberculosis* (Mtb), *M. leprae*, non-tuberculous species (NTM) pathogens, such as *M. chelonae*, *M. avium*, *M. fortuitum*, and *M. abscessus* and soil-saprophyte such as *M. smegmatis* (Msm) [35]. Among these diverse mycobacterial species, *M. tuberculosis* is a leading cause of mortality in humans as it causes tuberculosis (TB) [36]. The tubercle bacillus can not only infect but also persist within the host for several years. To establish the long-term infection, *M. tuberculosis* employs stringent response as one of its tools [24]. TB treatment consists of at least six months of antibiotic therapy [37]. However, the treatment may last up to two years in the case of drug-resistant TB. Drug-resistant TB also poses a serious threat to public health due to its contagious nature and spectrum of drug-resistance [8,38,39,40]. Hence, there is an urgent need to shorten the duration of TB treatment and contain the threat of drug-resistant TB. These two aims can be rapidly achieved by targeting the stringent response, which regulates persistence, drug resistance, and biofilm formation in several bacterial pathogens [16,41,42], though there is limited information on mycobacteria. Pathogenic and non-pathogenic mycobacteria express enzymes that metabolize (p)ppGpp [43]. Therefore, investigating the stringent response in all clinically relevant mycobacterial species is important. The majority of studies on mycobacterial stringent response have been carried out using either *M. tuberculosis* or *M. smegmatis* species. In this review, we present an overview of the stringent response in mycobacteria and describe the metabolism of all three alarmones—ppGpp, pppGpp, and the recently discovered pGpp (Figure 1).

We have also described how stringent response regulates long-term survival, pathogenesis, virulence, antibiotic resistance, and biofilm formation in *M. tuberculosis* and *M. smegmatis*. We have also delved into the literature pertaining to the chemical inhibition of stringent response in mycobacteria and made a case for (p)ppGpp analogs that can inhibit stringent response and can be used as antimycobacterial compounds.

## 2. Metabolism of (p)ppGpp in Mycobacteria

The genes encoding the enzymes for (p)ppGpp metabolism have been found in all sequenced bacterial genomes—except the phyla Chlamydiae, Verrucomicrobia, Planctomycetes, and a few obligate intracellular symbiotic bacterial species—which makes stringent response a nearly ubiquitous phenomenon in bacteria [43]. In Gram-negative bacteria, the stringent response is governed by two enzymes—RelA and SpoT. RelA, encoded by the *relA* gene, is a monofunctional synthetase responsible for the synthesis of (p)ppGpp. On the other hand, the bifunctional SpoT, encoded by the *spoT* gene, acts primarily as a hydrolase responsible for the degradation of (p)ppGpp. SpoT can also synthesize (p)ppGpp in response to stress, which does not activate RelA. In mycobacteria, the alarmones are synthesized and degraded by a dual-function enzyme, Rel [44]. It is believed that RelA and SpoT have evolved from the same ancestral Rel protein, and the hydrolase domain has been inactive in RelA. Thus, RelA, SpoT and Rel proteins have similar domain architecture and have been characterized as the RelA Spo Homology (RSH) superfamily of proteins [43].

Both pathogenic and non-pathogenic mycobacterial genomes have a *rel* gene, which encodes bifunctional Rel protein [43]. In *M. tuberculosis*, the gene *rv2583c* encodes this bifunctional Rel, which is a 790 amino acid long multidomain protein, comprising catalytic N-terminal domain (1–394 aa) and a regulatory C-terminal domain (395–790 aa). The N-terminal domain harbors both the hydrolase activity (1–181 aa) and the synthetase activity (87–394 aa) [43,45,46]. Amino acid residues 87–181 are shared between the hydrolase and synthetase activities of the N-terminal domain and form a three-helix bundle. Both the enzymatic activities require Mn^2+^ or Mg^2+^ cations as co-factors [27,44,46] (Figure 1A).

Several bacteria including mycobacteria also encode homologs of RSH proteins, which are smaller in length. These proteins are usually single-domain proteins and possess either synthetase or hydrolase activity. Hence, they are called small alarmone synthetases (SASs) or small alarmone hydrolases (SAH) [47,48,49,50]. The genomes of both *M. tuberculosis* and *M. smegmatis* contain one copy of SAS [30,47,51,52] (Figure 1A). In *M. tuberculosis*, the gene *rv1366* was predicted to encode a potential SAS. However, the Rv1366 protein was shown to be catalytically inactive as it could not synthesize (p)ppGpp [51]. The saprophytic *M. smegmatis* encodes RelZ, a SAS protein, which can synthesize the third alarmone, pGpp, unlike its *M. tuberculosis* ortholog Rv1366 (Figure 1). RelZ protein also possesses N-terminal RNase HII domain, which removes RNA–DNA hybrids generated during DNA replication [52]. The presence of both RNase HII and pGpp synthetase domains is needed for the RelZ catalytic activity, as individual domains have been shown to be enzymatically inactive. The catalytic inactivation of one domain does not impair the enzymatic activity of the other domain [52]. RelZ prefers GMP as substrate, unlike Rel_Mtb_, which prefers GDP/GTP as the substrate. Although no SAH has been identified in mycobacteria, Rel_Msm_ cleaves pGpp to GMP and pyrophosphate [30] (Figure 1B).

## 3. Mycobacterial Stringent Response and Its Role in Survival during Stress

Alarmones (p)ppGpp control cellular processes by affecting DNA replication, transcription, and translation, and thus, bring about timely changes in bacterial physiology. In this regard, there are excellent reviews that provide detailed information on other bacterial species such as *E. coli* [26,53,54,55,56,57,58,59]. *M. tuberculosis* faces several stresses, such as oxidative, nitrosative, and nutrient stress upon infecting the host. However, it overcomes these potentially fatal stresses and successfully establishes chronic infection [38,60,61]. The adaptation to such stresses requires a large-scale transcriptional reprogramming, which eventually lets *M. tuberculosis* not only infect the macrophages but also survive for years in granuloma [38,61,62].

The hallmark of stringent response is downregulation of rRNA and ribosomal protein synthesis with concomitant upregulation of amino acid biosynthetic operons to supply necessary amino acids for survival [63,64]. The *M. tuberculosis* strain H37Rv, like other bacteria, also shows these signature transcriptional changes upon nutrient deprivation. Microarray analysis of H37Rv and H37RvΔ*rel_Mtb_* mutant strains, starved for six hours, showed differential expression of several genes [65]. The study found that 54 genes encoding ribosomal proteins were downregulated in the parental H37Rv strain compared to H37RvΔ*rel_Mtb_* mutant. Moreover, the parental H37Rv strain also showed the downregulation of 5 genes involved in transcription and 16 genes involved in protein synthesis. Late-log phase cultures of H37RvΔ*rel_Mtb_* showed at least five-fold more ribosomes per unit protein when compared to the parental H37Rv strain. Thus, in the absence of (p)ppGpp, *M. tuberculosis* fails to regulate the synthesis rRNA and ribosomal protein, which are needed for adaptation to the stationary phase. This aspect of mycobacterial stringent response is similar to *E. coli*. However, some aspects of stringent response are mycobacteria specific such as CarD-based regulation and inorganic polyphosphate (polyP)-based regulation.

**Figure 2:**
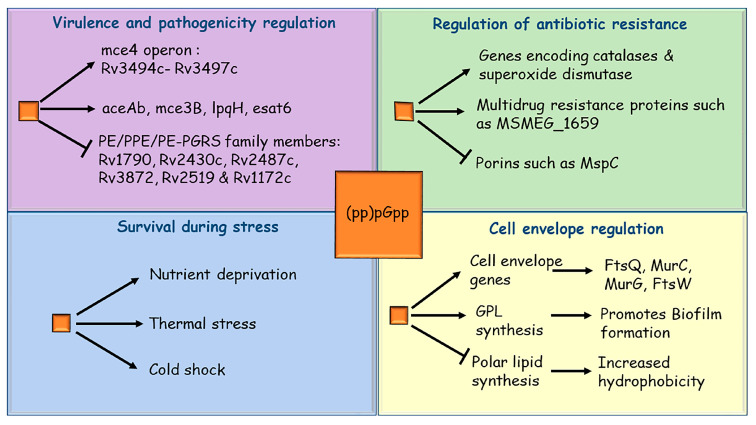
Pathways and genes affected by alarmones (pp)pGpp in mycobacteria: Alarmones (pp)pGpp (orange square) and associated Rel proteins regulate several processes in mycobacteria. Regulation of these processes results in the alteration of specific genes and their cognate pathways. The figure shows four major schemes—(i) virulence and pathogenicity, (ii) antibiotic resistance, (iii) stress, and (iv) cell envelope-related processes. The specific pathways and genes under these schemes have been depicted.

CarD is a conserved essential transcriptional regulator found in actinobacteria and its depletion in *M. tuberculosis* and *M. smegmatis* impaired the stringent response [66]. Stringent response in mycobacteria is also regulated by a feedback loop between (p)ppGpp and inorganic polyphosphate [67,68,69,70]. PolyP is synthesized and degraded by polyphosphate kinases (PPK) and exopolyphosphatases (PPX), respectively. A signaling cascade between two-component system MprAB, alternative sigma factor SigE and Rel protein governs the levels of (p)ppGpp and polyP in mycobacteria. PolyP functions as a phosphate donor to MprB, a stress-responsive histidine kinase. Subsequently, the phosphorylated MprB transfers its phosphoryl group to MprA, a response regulator. The phosphorylated MprA then activates the transcription of alternative sigma factor SigE, which eventually upregulates the transcription of *relA* gene [68,71,72,73]. Thus, apart from carbon starvation, nutrient starvation and hypoxia, phosphate starvation can also trigger the stringent response in mycobacteria. In the absence of stringent response, the long-term survival of both *M. tuberculosis* and *M. smegmatis* during stress conditions is impaired [24,74,75]. H37RvΔ*rel_Mtb_* mutant exhibited a slower growth rate than the parental H37Rv *M. tuberculosis* in synthetic media and failed to survive long-term starvation (4 months). Moreover, the H37RvΔ*rel_Mtb_* mutant failed to survive the oxygen limitation and increased temperature of 42 °C, and lost viability sooner than the parental *M. tuberculosis* H37Rv strain [74]. A subsequent study showed that H37RvΔ*rel_Mtb_* has very low levels of heat-shock protein HspX, which is needed for the adaptation to heat shock, and its low expression explains the inability of H37RvΔ*rel_Mtb_* mutant to grow at 42 °C [76]. Thus, the presence of Rel offers a survival advantage to *M. tuberculosis* during stress conditions. Similarly, in *M. smegmatis*, the deletion of the *rel* gene compromises long-term survival during nutrient starvation or grows slowly when subjected to cold shock [42,75]. Taken together, stringent response is important in both *M. tuberculosis* and *M. smegmatis* for the adaptation to stress conditions; otherwise, mycobacteria cannot survive under these hostile conditions in the host or environment (Figure 2).

## 4. Stringent Response Regulates Mycobacterial Virulence

Most of the TB infections remain asymptomatic as *M. tuberculosis* can successfully establish a latent chronic infection. It has been shown that the virulence of *M. tuberculosis* is also regulated by the stringent response [65,77]. The H37RvΔ*rel_Mtb_* mutant can establish an infection in the mice model and during the first few weeks post-infection, its growth is indistinguishable from the parental H37Rv strain. However, after five weeks of infection, the viability of the mutant starts dropping, and four months post-infection, the bacterial load in both lungs and spleen is 500-fold lower than the parental *M. tuberculosis* H37Rv [65]. The lungs of mice infected with the parental strain showed the presence of several granulomas, which covered almost one-third of lung tissue. In contrast, lungs infected with H37RvΔ*rel_Mtb_* strain showed significantly fewer granulomas, and almost normal lung architecture [65]. Thus, stringent response is necessary for the maintenance of chronic *M. tuberculosis* infection.

Transcriptomic analysis through microarray showed that several genes associated with mycobacterial virulence and antigens were differentially expressed in H37RvΔ*rel_Mtb_* mutant compared to the parental H37Rv strain [65]. These included *groEL2* and *groES*, the 19-kDa antigen LpqH, and members of the *PE_PGRS* family. Secreted antigens such as *esat6*, the antigen 85 complex, *mpt83*, and *cfp7* were also found to be differentially expressed in H37RvΔ*rel_Mtb_* mutant. The expression of Lipoprotein LpqH decreases in H37RvΔ*rel_Mtb_* mutant. LpqH is known to inhibit cytokine secretion, decrease the antigen presentation to macrophage and promote macrophage apoptosis [78] (Figure 2). The PE_PGRS proteins modulate antigenic variation in clinical isolates of *M. tuberculosis* [79,80]. Moreover, these proteins also contribute to the survival of *M. tuberculosis* in granuloma [81]. Recently, it was shown that PE_PGRS3, localized on the mycobacterial cell surface, is expressed during phosphate limitation, a condition which also triggers the stringent response in *M. tuberculosis* and *M. smegmatis* [82]. The C-terminal of PE_PGRS3 is arginine-rich and, hence, positively charged. This helps *M. tuberculosis* to establish contact with negatively charged phospholipids on the host cell membrane. The expression of PE_PGRS3, which is dependent on (p)ppGpp, is needed for interaction between *M. tuberculosis* and the host cell, and to eventually establish the infection [83]. Thus, at the molecular level, stringent response regulates mycobacterial persistence inside the host by modulating the expression of important genes involved in virulence and antigen presentation.

The effect of stringent response on mycobacterial survival has also been assessed in guinea pigs, as the granulomas in guinea pigs resemble those found in humans in terms of architecture, composition, and caseation necrosis [77]. There was a reduced burden in the lungs of guinea pigs infected with H37RvΔ*rel_Mtb_* strain compared with the parental H37Rv strain, which indicated that impairment of the stringent response reduces *M. tuberculosis* survival during infection in lungs. Moreover, the guinea pig lungs infected with the H37RvΔ*rel_Mtb_* strain showed considerably fewer tubercle lesions and caseous granulomas. Thus, in the absence of the stringent response, *M. tuberculosis* cannot establish a chronic infection, as (p)ppGpp is needed for the expression of key virulence proteins and maintenance of long-term infection inside the host (Figure 2).

## 5. Role of Stringent Response in Mycobacterial Drug Resistance

Bacterial infections are treated through antibiotic therapy. However, the indiscriminate use of antibiotics has led to the rise of several drug-resistant bacteria, and mycobacteria are no exception. The rise of multidrug-resistant (MDR-TB) and extensively drug-resistant (XDR-TB) bacteria is now posing a serious threat to public health worldwide [84,85,86]. Stringent response has also been shown to regulate antibiotic resistance in different bacteria. *E. coli* subjected to amino acid starvation show resistance to β-lactam antibiotics [87,88,89]. This was later corroborated when various strains of *E. coli* defective in (p)ppGpp signaling were found to be sensitive to a wide range of antimicrobial compounds [90]. Furthermore, activation of the stringent response—and thus, an increase in (p)ppGpp levels—are associated with antibiotic tolerance in *Pseudomonas aeruginosa* [91]. We will discuss a few studies which show the relationship between stringent response, regulators of stringent response and antibiotic susceptibility in mycobacteria.

Recently, it has been shown that the *M. tuberculosis* strain deficient of Rel has altered metabolism and loses the ability to become quiescent [92]. Further, inhibitors targeting Rel not only kill *M. tuberculosis* but also enhance the potency of isoniazid. These results show the importance of the stringent response in persistence, and its therapeutic importance to develop new drugs. The relationship between stringent response and antibiotic susceptibility in *M. smegmatis* has been studied using high-throughput phenotype microarray technology [41,93]. The phenotype microarray analyses showed that the Δ*rel_Msm_* strain was resistant to multiple antibiotics compared to the parental mc^2^155 *M. smegmatis*. The results of phenotype microarray were subsequently verified by determining the minimum inhibitory concentration (MIC) of representative antibiotics using the broth microdilution assay. The Δ*rel_Msm_* strain showed increased resistance to rifampicin in both MIC-based assay and phenotype microarray [41,94]. However, the exact cause of increased resistance to rifampicin by Δ*rel_Msm_* could not be deciphered. It was proposed that changes in cell wall lipid compositions of Δ*rel_Msm_* might have hindered the uptake of rifampicin, thus contributing to increased rifampicin resistance [41]. Increased expression of genes encoding several multidrug resistance-associated proteins that encode catalases and superoxide dismutase was also proposed to be a possible cause of resistance shown by Δ*rel_Msm_* to rifampicin and other antibiotics [42,65] (Figure 2). Additionally, the qRT-PCR analysis of Δ*rel_Msm_* showed down-regulation of porins, which might contribute to its multidrug resistance [65].

Interestingly, the Δ*rel_Msm_* strain is not a (p)ppGpp null mutant strain, as *M. smegmatis* also encodes RelZ, a small alarmone synthase [47,52]. To elucidate the role of RelZ, the SAS in *M. smegmatis*, the antibiotic sensitivity profile *relZ* knockout was studied. Unlike the Δ*rel_Msm_* strain, the Δ*relZ* strain was sensitive to several antibiotics such as bleomycin, ofloxacin, and rifampin. The double knockout strain Δ*rel_Msm_*Δ*relZ* showed an antibiotic sensitivity profile similar to that of the Δ*relZ* strain [30]. Thus, it appears that in *M. smegmatis*, the relationship between (pp)pGpp levels and antibiotic resistance is more complex than other bacteria. Moreover, the Δ*rel_Msm_*Δ*relZ* strain is also not a (p)ppGpp null mutant strain, as it is predicted to possess another (pp)pGpp synthase. Hence, to completely decipher the role of (pp)pGpp in antibiotic resistance in *M. smegmatis*, a (pp)pGpp null strain would be helpful.

## 6. Stringent Response and Biofilm Formation

Biofilms are structured communities of bacteria embedded in self-produced polymeric matrices and attached to an abiotic or living surface [95,96]. Biofilms protect bacteria from antibiotics, host immune system, and other environmental insults; they are a common cause of persistent bacterial infection [95,97]. NTMs or environmental mycobacteria have been shown to form biofilms. These include *M.* *chelonae*, *M. avium*, *M. fortuitum*, *M.* *abscessus* and *M. smegmatis* [42,98,99,100,101,102]. These mycobacterial biofilms have been found in both natural and manufactured settings such as soil, showerheads, hospital water system and medical equipment. NTMs cause skin and soft tissue infections, aseptic meningitis, lymphadenitis, and pulmonary infections [103,104,105]. Disseminated and mixed NTM infections have been found in immunocompromised individuals suffering from cystic fibrosis, renal failures, leukemia and organ transplant recipients [106]. The prevalence of *M. abscessus* in patients with chronic lung infections is also rising steadily [107,108]. *M. tuberculosis* has also been shown to form biofilms both in vitro and in vivo [109,110,111]. The mycobacterial biofilm matrix comprises extracellular DNA, carbohydrates, lipids and proteins [112,113]. Biofilm formation, like a stringent response, is a way to survive in harsh or unfavorable environments. Hence, it is likely that stringent response might also regulate biofilm formation in mycobacteria. In the last two decades, the role of (p)ppGpp in the regulation of biofilm formation, particularly in pathogenic bacteria, is becoming well understood. For example, in *Listeria monocytogenes*, deletion of *relA* impairs biofilm formation and reduces virulence [114]. In *Streptococcus mutans*, *relA* inactivation causes a reduction in biofilm formation capacity [115]. *Enterococcus faecalis* lacking (p)ppGpp show diminished capacity to form biofilms [116]. In *Vibrio cholerae*, the inactivation of stringent response results in reduced ability to form biofilms [117]. In the ppGpp null mutant strain of *P. aeruginosa*, the biofilms cells are more sensitive to antibiotics compared with the cells from wild-type biofilms [91].

Given the important role stringent response plays in biofilm formation in several bacterial pathogens, its role in mycobacterial biofilm formation has also been investigated. In *M. smegmatis*, the stringent response has been shown to control biofilm formation [41] as the Δ*rel_Msm_* strain is deficient in biofilm formation, has reduced sliding motility and rough colony morphology. These phenotypes are governed by glycopeptidolipids (GPLs), which are a peculiar class of lipids, found in NTMs and *M. smegmatis*, and are needed for biofilm formation [118]. The Δ*rel_Msm_* strain has reduced levels of GPLs in its cell wall compared to the parental mc^2^155 strain. This is indicative of the fact that the stringent response may regulate biofilm formation and colony morphology in *M. smegmatis* by regulating the synthesis of GPLs (Figure 2). Moreover, both Δ*rel_Mtb_* and Δ*rel_Msm_* strains also exhibit differential expression of several genes involved in cell envelope biosynthesis [42,65,119]. The deletion of small alarmone synthetase, RelZ, also impaired the biofilm formation in *M. smegmatis*. However, the degree of impairment is not as strong as that seen for the Δ*rel_Msm_* strain. Moreover, the double knockout Δ*rel*Δ*relZ* of *M. smegmatis* shows the strongest inhibition of biofilm formation [30]. Based on these observations, it seems that Rel_Msm_ is the principal mediator of stringent response, while RelZ has a relatively minor contribution in this process. Thus, the stringent response regulates biofilm in mycobacteria by regulating the expression of genes involved in GPL and other cell wall components. Since NTMs utilize biofilm formation to establish infections, given their ability to form biofilms on medical implants and water distribution systems, it is very important to also explore the role of stringent response in NTM species [105,120,121].

## 7. Chemical Inhibition of Stringent Response as a Therapeutic Tool

Since the stringent response is important for several processes, such as persistence, virulence, antibiotic resistance, and biofilm formation, its chemical inhibition might be an attractive way to address the problem of drug resistance. In this direction, relacin, a synthetic (p)ppGpp analog, has been shown to inhibit stringent response in *B. subtilis* and *B. anthracis*. Relacin binds to Rel protein near the active site, which inhibits (p)ppGpp synthesis [122] and inhibits stringent response both in vivo and in vitro. Moreover, it also blocks the sporulation process in both *B. subtilis* and *B. anthracis*, when added to sporulating cultures, irrespective of the stage of sporulation. Since relacin blocks (p)ppGpp synthesis, it also inhibits biofilm formation in *Bacillus* species. In an alternative approach, an anti-biofilm peptide, 1018, was found to interact with (p)ppGpp [123]. The peptide was identified in a screen and was previously labeled as an innate defense regulator due to its immunomodulatory activities; 1018 was able to degrade (p)ppGpp, the mediator of stringent response in several clinically important species. These include pathogens such as *P. aeruginosa*, *E. coli*, *Acinetobacter baumannii*, *Klebsiella pneumoniae*, methicillin-resistant *Staphylococcus aureus*, *Salmonella typhimurium*, and *Burkholderia cenocepacia*, which when treated with 1018, failed to form biofilms. A low dosage of 1018 triggered biofilm dispersal, while the high dosage caused the death of bacterial cells in the biofilms. Additionally, overproduction of (p)ppGpp in *P. aeruginosa* and *S. aureus* imparted resistance to 1018 [123]. These studies demonstrated that chemical inhibition of stringent response can be used to inhibit pathogenic bacteria.

Recently, such approaches have also been applied to inhibit mycobacterial stringent response [124,125]. In one such study, acetylated and benzoylated (p)pGpp—N2,2′,3′,5′-O-Tetraacetylguanosine and N2,2′-O,3′-O,5′-O-Tetrabenzoylguanosine—were synthesized to assess if these compounds can inhibit stringent response in mycobacteria. Both the compounds significantly inhibited the activity of Rel_Msm_ protein in vitro and in vivo. These compounds also inhibited biofilm formation in both *M. smegmatis* and *M. tuberculosis*. Moreover, these compounds were not toxic in cell culture assays, thus demonstrating a potential to be used in in vivo studies in mice. Furthermore, vitamin C was also shown to be used as a chemical inhibitor of stringent response in *M. smegmatis* [126]. Vitamin C-treated *M. smegmatis* cultures show lower levels of (p)ppGpp compared to untreated control. Vitamin C also inhibited the activity of Rel_Msm,_ possibly leading to decreased synthesis of (p)ppGpp. Interestingly, treatment with vitamin C also inhibited biofilm formation by *M. smegmatis*. However, whether the inhibition of stringent response by vitamin C is responsible for the disruption of biofilm formation remains to be elucidated. In another study, a chemically synthesized compound called DMNP [4-(4,7-DiMethyl-1,2,3,4-tetrahydroNaphthalene-1-yl)] Pentanoic acid—an analog of natural marine diterpene erogorgiaene—could bind to Rel_Msm_ protein and inhibit its (p)ppGpp synthase activity. Moreover, when *M. smegmatis* cultures were treated with DMNP, they failed to form biofilms and their persistence was reduced [124]. Hence, these studies demonstrate that inhibition of stringent response is an attractive target to design novel antimycobacterial compounds.

## 8. Outlook

The stringent response is an important survival strategy used by both non-pathogenic and pathogenic bacteria. The mycobacterial genus includes important human pathogens such as *M. tuberculosis*. *M. leprae*, *M. ulcerans* and opportunistic pathogens such as *M. avium*, *M. fortuitum*, and *M. abscessus*. However, unlike other bacterial pathogens, the stringent response remains an underexplored area for mycobacterial species. Given that the stringent response regulates important processes of persistence, virulence, drug resistance, and biofilm formation, its molecular mechanism would be important to study in all clinically relevant mycobacterial species. Since mycobacterial species occupy diverse niches, it is likely that some aspects of stringent response can be species-specific. Based on the current literature, it can be proposed that the conserved Rel protein is likely the principal mediator of stringent response in all mycobacterial species, while the species-specific differences in stringent response could be modulated through small alarmone synthetases and small alarmone hydrolases. The chemical inhibition of stringent response in *M. smegmatis* and *M. tuberculosis* by the same compounds points to the conserved nature of Rel-mediated (p)ppGpp signaling [125]. On the other hand, small alarmone synthetases RelZ from *M. smegmatis* and Rv1366 from *M. tuberculosis* show species-specific differences [30,51]. With the recent advancement in tools, it is possible to identify specific targets of alarmones. For example, using photo-cross-linkable (p)ppGpp, its targets can be identified precisely. Using this technique, it is possible to elaborate the molecular details of stringent response in a species-specific manner [127]. Recently, an RNA-based fluorescent sensor for live-cell imaging of (p)ppGpp was developed [128]. It can be applied to study (p)ppGpp dynamics in real-time in mycobacteria, which may help unravel the interaction partners of (p)ppGpp. Given the rise of antibiotic resistance and its close association with alarmones, it is important to investigate the stringent response across all mycobacterial species.

## Data Availability

Not applicable.

## References

[1] Arora G., Misra R., Sajid A. (2017). Model Systems for Pulmonary Infectious Diseases: Paradigms of Anthrax and Tuberculosis. Curr. Top. Med. Chem..

[2] Konstantinidis K.T., Viver T., Conrad E.R., Venter S.N., Rossello-Mora R. (2021). Solar salterns as model systems to study the units of bacterial diversity that matter for ecosystem functioning. Curr. Opin. Biotechnol..

[3] Liu H., Deutschbauer A.M. (2018). Rapidly moving new bacteria to model-organism status. Curr. Opin. Biotechnol..

[4] Mehta P., Ray A., Mazumder S. (2021). TLRs in Mycobacterial Pathogenesis: Black and White or Shades of Gray. Curr. Microbiol..

[5] Bellinzoni M., Wehenkel A.M., Durán R., Alzari P.M. (2019). Novel mechanistic insights into physiological signaling pathways mediated by mycobacterial Ser/Thr protein kinases. Genes Immun..

[6] Virmani R., Sajid A., Singhal A., Gaur M., Joshi J., Bothra A., Garg R., Misra R., Singh V.P., Molle V. (2019). The Ser/Thr protein kinase PrkC imprints phenotypic memory in Bacillus anthracis spores by phosphorylating the glycolytic enzyme enolase. J. Biol. Chem..

[7] Arora G., Sajid A., Singhal A., Joshi J., Virmani R., Gupta M., Verma N., Maji A., Misra R., Baronian G. (2014). Identification of Ser/Thr kinase and Forkhead Associated Domains in Mycobacterium ulcerans: Characterization of Novel Association between Protein Kinase Q and MupFHA. PLoS Negl. Trop. Dis..

[8] Arora G., Bothra A., Prosser G., Arora K., Sajid A. (2020). Role of post-translational modifications in the acquisition of drug resistance in *Mycobacterium tuberculosis*. FEBS J..

[9] Romaní-Pérez M., Bullich-Vilarrubias C., López-Almela I., Liébana-García R., Olivares M., Sanz Y. (2021). The Microbiota and the Gut–Brain Axis in Controlling Food Intake and Energy Homeostasis. Int. J. Mol. Sci..

[10] Papon N., Stock A.M. (2019). What do archaeal and eukaryotic histidine kinases sense?. F1000Research.

[11] Arora G., Sajid A., Gupta M., Bhaduri A., Kumar P., Basu-Modak S., Singh Y. (2010). Understanding the Role of PknJ in *Mycobacterium tuberculosis*: Biochemical Characterization and Identification of Novel Substrate Pyruvate Kinase A. PLoS ONE.

[12] Sajid A., Arora G., Gupta M., Singhal A., Chakraborty K., Nandicoori V.K., Singh Y. (2011). Interaction of *Mycobacterium tuberculosis* Elongation Factor Tu with GTP Is Regulated by Phosphorylation. J. Bacteriol..

[13] Hauryliuk V., Atkinson G., Murakami K.S., Tenson T., Gerdes K. (2015). Recent functional insights into the role of (p) ppGpp in bacterial physiology. Nat. Rev. Genet..

[14] Potrykus K., Cashel M. (2008). (p)ppGpp: Still Magical?. Annu. Rev. Microbiol..

[15] Srivatsan A., Wang J.D. (2008). Control of bacterial transcription, translation and replication by (p)ppGpp. Curr. Opin. Microbiol..

[16] Dalebroux Z.D., Svensson S.L., Gaynor E.C., Swanson M.S. (2010). ppGpp Conjures Bacterial Virulence. Microbiol. Mol. Biol. Rev..

[17] Pacios O., Blasco L., Bleriot I., Fernandez-Garcia L., Ambroa A., López M., Bou G., Cantón R., Garcia-Contreras R., Wood T.K. (2020). (p)ppGpp and Its Role in Bacterial Persistence: New Challenges. Antimicrob. Agents Chemother..

[18] Cashel M., Gallant J. (1969). Two Compounds implicated in the Function of the RC Gene of *Escherichia coli*. Nature.

[19] Stent G.S., Brenner S. (1961). A Genetic Locus for the Regulation of Ribonucleic Acid Synthesis. Proc. Natl. Acad. Sci. USA.

[20] Wagner R. (2002). Regulation of ribosomal RNA synthesis in E. coli: Effects of the global regulator guanosine tetraphosphate (ppGpp). J. Mol. Microbiol. Biotechnol..

[21] Paul B.J., Ross W., Gaal T., Gourse R.L. (2004). rRNA Transcription in *Escherichia coli*. Annu. Rev. Genet..

[22] Lamond A., Travers A.A. (1985). Stringent control of bacterial transcription. Cell.

[23] Chatterji D., Ojha A.K. (2001). Revisiting the stringent response, ppGpp and starvation signaling. Curr. Opin. Microbiol..

[24] Primm T.P., Andersen S.J., Mizrahi V., Avarbock D., Rubin H., Barry C.E. (2000). The Stringent Response of Mycobacterium tuberculosis Is Required for Long-Term Survival. J. Bacteriol..

[25] Ojha A.K., Mukherjee T.K., Chatterji D. (2000). High Intracellular Level of Guanosine Tetraphosphate in *Mycobacterium smegmatis* Changes the Morphology of the Bacterium. Infect. Immun..

[26] Liu K., Bittner A.N., Wang J.D. (2015). Diversity in (p) ppGpp metabolism and effectors. Curr. Opin. Microbiol..

[27] Jain V., Saleem-Batcha R., China A., Chatterji D. (2006). Molecular dissection of the mycobacterial stringent response protein Rel. Protein Sci..

[28] Boutte C., Crosson S. (2013). Bacterial lifestyle shapes stringent response activation. Trends Microbiol..

[29] Yang J., Anderson B.W., Turdiev A., Turdiev H., Stevenson D.M., Amador-Noguez D., Lee V.T., Wang J.D. (2020). The nucleotide pGpp acts as a third alarmone in Bacillus, with functions distinct from those of (p) ppGpp. Nat. Commun..

[30] Petchiappan A., Naik S.Y., Chatterji D. (2020). RelZ-Mediated Stress Response in *Mycobacterium smegmatis*: pGpp Synthesis and Its Regulation. J. Bacteriol..

[31] Irving S.E., Choudhury N.R., Corrigan R.M. (2020). The stringent response and physiological roles of (pp) pGpp in bacteria. Nat. Rev. Genet..

[32] Ooga T., Ohashi Y., Kuramitsu S., Koyama Y., Tomita M., Soga T., Masui R. (2009). Degradation of ppGpp by Nudix Pyrophosphatase Modulates the Transition of Growth Phase in the Bacterium *Thermus thermophilus*. J. Biol. Chem..

[33] Krishnan S., Chatterji D. (2020). Pleiotropic Effects of Bacterial Small Alarmone Synthetases: Underscoring the Dual-Domain Small Alarmone Synthetases in *Mycobacterium smegmatis*. Front. Microbiol..

[34] Gaca A.O., Kudrin P., Winter C.C., Beljantseva J., Liu K., Anderson B., Wang J., Rejman D., Potrykus K., Cashel M. (2015). From (p)ppGpp to (pp)pGpp: Characterization of Regulatory Effects of pGpp Synthesized by the Small Alarmone Synthetase of *Enterococcus faecalis*. J. Bacteriol..

[35] Forbes B.A. (2017). Mycobacterial Taxonomy. J. Clin. Microbiol..

[36] Furin J., Cox H., Pai M. (2019). Tuberculosis. Lancet.

[37] Gupta V.K., Kumar M.M., Singh D., Bisht D., Sharma S. (2017). Drug targets in dormant *Mycobacterium tuberculosis*: Can the conquest against tuberculosis become a reality?. Infect. Dis..

[38] Pai M., Behr M.A., Dowdy D., Dheda K., Divangahi M., Boehme C.C., Ginsberg A., Swaminathan S., Spigelman M., Getahun H. (2016). Tuberculosis. Nat. Rev. Dis. Prim..

[39] Lange C., Dheda K., Chesov D., Mandalakas A.M., Udwadia Z., Horsburgh C.R. (2019). Management of drug-resistant tuberculosis. Lancet.

[40] Lange C., Chesov D., Heyckendorf J., Leung C.C., Udwadia Z., Dheda K. (2018). Drug-resistant tuberculosis: An update on disease burden, diagnosis and treatment. Respirology.

[41] Gupta K.R., Kasetty S., Chatterji D. (2015). Novel Functions of (p)ppGpp and Cyclic di-GMP in Mycobacterial Physiology Revealed by Phenotype Microarray Analysis of Wild-Type and Isogenic Strains of *Mycobacterium smegmatis*. Appl. Environ. Microbiol..

[42] Gupta K.R., Baloni P., Indi S.S., Chatterji D. (2016). Regulation of Growth, Cell Shape, Cell Division, and Gene Expression by Second Messengers (p)ppGpp and Cyclic Di-GMP in *Mycobacterium smegmatis*. J. Bacteriol..

[43] Atkinson G.C., Tenson T., Hauryliuk V. (2011). The RelA/SpoT Homolog (RSH) Superfamily: Distribution and Functional Evolution of ppGpp Synthetases and Hydrolases across the Tree of Life. PLoS ONE.

[44] Prusa J., Zhu D., Stallings C.L. (2018). The stringent response and *Mycobacterium tuberculosis* pathogenesis. Pathog. Dis..

[45] Sajish M., Kalayil S., Verma S.K., Nandicoori V.K., Prakash B. (2009). The significance of EXDD and RXKD motif conservation in Rel proteins. J. Biol. Chem..

[46] Avarbock D., Salem J., Li L.-S., Wang Z.-M., Rubin H. (1999). Cloning and characterization of a bifunctional RelA/SpoT homologue from Mycobacterium tuberculosis. Gene.

[47] Murdeshwar M.S., Chatterji D. (2012). MS_RHII-RSD, a Dual-Function RNase HII-(p) ppGpp Synthetase from *Mycobacterium smegmatis*. J. Bacteriol..

[48] Lemos J.A., Lin V.K., Nascimento M.M., Abranches J., Burne R.A. (2007). Three gene products govern (p)ppGpp production by Streptococcus mutans. Mol. Microbiol..

[49] Das B., Pal R.R., Bag S., Bhadra R.K. (2009). Stringent response in Vibrio cholerae: Genetic analysis of spoT gene function and identification of a novel (p) ppGpp synthetase gene. Mol. Microbiol..

[50] Abranches J., Martinez A.R., Kajfasz J.K., Chaávez V., Garsin D.A., Lemos J.A. (2009). The Molecular Alarmone (p)ppGpp Mediates Stress Responses, Vancomycin Tolerance, and Virulence in *Enterococcus faecalis*. J. Bacteriol..

[51] Weiss L.A., Stallings C.L. (2013). Essential Roles for *Mycobacterium tuberculosis* Rel beyond the Production of (p) ppGpp. J. Bacteriol..

[52] Krishnan S., Petchiappan A., Singh A., Bhatt A., Chatterji D. (2016). R-loop induced stress response by second (p)ppGpp synthetase in *Mycobacterium smegmatis*: Functional and domain interdependence. Mol. Microbiol..

[53] Laurie A.D., Bernardo L.M.D., Sze C.C., Skärfstad E., Szalewska-Palasz A., Nyström T., Shingler V. (2003). The Role of the Alarmone (p)ppGpp in ςN Competition for Core RNA Polymerase. J. Biol. Chem..

[54] Jishage M., Kvint K., Shingler V., Nyström T. (2002). Regulation of sigma factor competition by the alarmone ppGpp. Genes Dev..

[55] Ramnaresh Gupta K., Chatterji D., de Bruijn F.J. (2016). Sigma Factor Competition in *Escherichia Coli*: Kinetic and Thermodynamic Perspectives. Stress and Environmental Regulation of Gene Expression and Adaptation in Bacteria.

[56] Rasouly A., Pani B., Nudler E. (2016). A Magic Spot in Genome Maintenance. Trends Genet..

[57] Szalewska-Palasz A., Wegrzyn G., Wegrzyn A. (2007). Mechanisms of physiological regulation of RNA synthesis in bacteria: New discoveries breaking old schemes. J. Appl. Genet..

[58] Anderson B.W., Fung D.K., Wang J.D. (2021). Regulatory Themes and Variations by the Stress-Signaling Nucleotide Alarmones (p) ppGpp in Bacteria. Annu. Rev. Genet..

[59] Milon P., Tischenko E., Tomšic J., Caserta E., Folkers G., La Teana A., Rodnina M., Pon C.L., Boelens R., Gualerzi C.O. (2006). The nucleotide-binding site of bacterial translation initiation factor 2 (IF2) as a metabolic sensor. Proc. Natl. Acad. Sci. USA.

[60] Stallings C.L., Glickman M.S. (2010). Is *Mycobacterium tuberculosis* stressed out? A critical assessment of the genetic evidence. Microbes Infect..

[61] Misra R., Menon D., Arora G., Virmani R., Gaur M., Naz S., Jaisinghani N., Bhaduri A., Bothra A., Maji A. (2019). Tuning the *Mycobacterium tuberculosis* Alternative Sigma Factor SigF through the Multidomain Regulator Rv1364c and Osmosensory Kinase Protein Kinase D. J. Bacteriol..

[62] Barry E.C., Boshoff H.I., Dartois V., Dick T., Ehrt S., Flynn J., Schnappinger D., Wilkinson R., Young D. (2009). The spectrum of latent tuberculosis: Rethinking the biology and intervention strategies. Nat. Rev. Genet..

[63] Traxler M.F., Summers S.M., Nguyen H.-T., Zacharia V.M., Hightower G.A., Smith J.T., Conway T. (2008). The global, ppGpp-mediated stringent response to amino acid starvation in *Escherichia coli*. Mol. Microbiol..

[64] Eymann C., Homuth G., Scharf C., Hecker M. (2002). Bacillus subtilis functional genomics: Global characterization of the stringent response by proteome and transcriptome analysis. J. Bacteriol..

[65] Dahl J.L., Kraus C.N., Boshoff H.I.M., Doan B., Foley K., Avarbock D., Kaplan G., Mizrahi V., Rubin H., Barry C.E. (2003). The role of RelMtb-mediated adaptation to stationary phase in long-term persistence of *Mycobacterium tuberculosis* in mice. Proc. Natl. Acad. Sci. USA.

[66] Stallings C.L., Stephanou N.C., Chu L., Hochschild A., Nickels B.E., Glickman M.S. (2009). CarD Is an Essential Regulator of rRNA Transcription Required for *Mycobacterium tuberculosis* Persistence. Cell.

[67] Sureka K., Ghosh B., Dasgupta A., Basu J., Kundu M., Bose I. (2008). Positive Feedback and Noise Activate the Stringent Response Regulator Rel in Mycobacteria. PLoS ONE.

[68] Sureka K., Dey S., Datta P., Singh A.K., Dasgupta A., Rodrigue S., Basu J., Kundu M. (2007). Polyphosphate kinase is involved in stress-induced mprAB-sigE-rel signalling in mycobacteria. Mol. Microbiol..

[69] Rifat D., Bishai W.R., Karakousis P.C. (2009). Phosphate Depletion: A Novel Trigger for *Mycobacterium tuberculosis* Persistence. J. Infect. Dis..

[70] Singh R., Singh M., Arora G., Kumar S., Tiwari P., Kidwai S. (2013). Polyphosphate Deficiency in Mycobacterium tuberculosis Is Associated with Enhanced Drug Susceptibility and Impaired Growth in Guinea Pigs. J. Bacteriol..

[71] Chuang Y.-M., Dutta N.K., Hung C.-F., Wu T.-C., Rubin H., Karakousis P.C. (2016). Stringent Response Factors PPX1 and PPK2 Play an Important Role in *Mycobacterium tuberculosis* Metabolism, Biofilm Formation, and Sensitivity to Isoniazid In Vivo. Antimicrob. Agents Chemother..

[72] Chuang Y.-M., Belchis D.A., Karakousis P.C. (2013). The Polyphosphate Kinase Gene *ppk2* is Required for *Mycobacterium tuberculosis* Inorganic Polyphosphate Regulation and Virulence. mBio.

[73] Chuang Y.-M., Bandyopadhyay N., Rifat D., Rubin H., Bader J.S., Karakousis P.C. (2015). Deficiency of the Novel Exopolyphosphatase Rv1026/PPX2 Leads to Metabolic Downshift and Altered Cell Wall Permeability in *Mycobacterium tuberculosis*. mBio.

[74] Avarbock A., Avarbock D., Teh J.-S., Buckstein M., Wang A.Z.-M., Rubin H. (2005). Functional Regulation of the Opposing (p)ppGpp Synthetase/Hydrolase Activities of RelMtb from *Mycobacterium tuberculosis*. Biochemistry.

[75] Mathew R., Ojha A.K., Karande A.A., Chatterji D. (2004). Deletion of the rel gene in *Mycobacterium smegmatis* reduces its stationary phase survival without altering the cell-surface associated properties. Curr. Sci..

[76] Dahl J.L., Arora K., Boshoff H.I., Whiteford D.C., Pacheco S.A., Walsh O.J., Lau-Bonilla D., Davis W.B., Garza A.G. (2005). The relA Homolog of *Mycobacterium smegmatis* Affects Cell Appearance, Viability, and Gene Expression. J. Bacteriol..

[77] Klinkenberg L.G., Lee J.-H., Bishai W.R., Karakousis P.C. (2010). The Stringent Response Is Required for Full Virulence of *Mycobacterium tuberculosis* in Guinea Pigs. J. Infect. Dis..

[78] Post F.A., Manca C., Neyrolles O., Ryffel B., Young D.B., Kaplan G. (2001). *Mycobacterium tuberculosis* 19-Kilodalton Lipoprotein Inhibits *Mycobacterium smegmatis*—Induced Cytokine Production by Human Macrophages In Vitro. Infect. Immun..

[79] Cole S.T., Brosch R., Parkhill J., Garnier T., Churcher C., Harris D., Gordon S., Eiglmeier K., Gas S., Barry E.C. (1998). Deciphering the biology of *Mycobacterium tuberculosis* from the complete genome sequence. Nature.

[80] Banu S., Honoré N., Saint-Joanis B., Philpott D., Prévost M.-C., Cole S.T. (2002). Are the PE-PGRS proteins of *Mycobacterium tuberculosis* variable surface antigens?. Mol. Microbiol..

[81] Ramakrishnan L., Federspiel N.A., Falkow S. (2000). Granuloma-Specific Expression of Mycobacterium Virulence Proteins from the Glycine-Rich PE-PGRS Family. Science.

[82] De Maio F., Battah B., Palmieri V., Petrone L., Corrente F., Salustri A., Palucci I., Bellesi S., Papi M., Rubino S. (2018). PE_PGRS3 of Mycobacterium tuberculosis is specifically expressed at low phosphate concentration, and its arginine-rich C-terminal domain mediates adhesion and persistence in host tissues when expressed in *Mycobacterium smegmatis*. Cell. Microbiol..

[83] De Maio F., Salustri A., Battah B., Palucci I., Marchionni F., Bellesi S., Palmieri V., Papi M., Kramarska E., Sanguinetti M. (2021). PE_PGRS3 ensures provision of the vital phospholipids cardiolipin and phosphatidylinositols by promoting the interaction between *M. tuberculosis* and host cells. Virulence.

[84] Singh V., Chibale K. (2021). Strategies to Combat Multi-Drug Resistance in Tuberculosis. Acc. Chem. Res..

[85] Seki M., Choi H.J., Kim K., Whang J., Sung J., Mitarai S. (2021). Tuberculosis: A persistent unpleasant neighbour of humans. J. Infect. Public Health.

[86] Allué-Guardia A., García J.I., Torrelles J.B. (2021). Evolution of Drug-Resistant *Mycobacterium tuberculosis* Strains and Their Adaptation to the Human Lung Environment. Front. Microbiol..

[87] Vinella D., D’Ari R., Jaffé A., Bouloc P. (1992). Penicillin binding protein 2 is dispensable in Escherichia coli when ppGpp synthesis is induced. EMBO J..

[88] Rodionov D.G., Ishiguro E.E. (1995). Direct correlation between overproduction of guanosine 3′,5′-bispyrophosphate (ppGpp) and penicillin tolerance in Escherichia coli. J. Bacteriol..

[89] Joseleau-Petit D., Thévenet D., D’Arl R. (1994). ppGpp concentration, growth without PBP2 activity, and growth-rate control in Escherichia coli. Mol. Microbiol..

[90] Greenway D.L.A., England R.R. (1999). The intrinsic resistance of *Escherichia coli* to various antimicrobial agents requires ppGpp and sigmas. Lett. Appl. Microbiol..

[91] Nguyen D., Joshi-Datar A., Lepine F., Bauerle E., Olakanmi O., Beer K., McKay G., Siehnel R., Schafhauser J., Wang Y. (2011). Active Starvation Responses Mediate Antibiotic Tolerance in Biofilms and Nutrient-Limited Bacteria. Science.

[92] Dutta N.K., Klinkenberg L.G., Vazquez M.-J., Segura-Carro D., Colmenarejo G., Ramon F., Rodriguez-Miquel B., Mata-Cantero L., Francisco E.P.-D., Chuang Y.-M. (2019). Inhibiting the stringent response blocks *Mycobacterium tuberculosis* entry into quiescence and reduces persistence. Sci. Adv..

[93] Baloni P., Padiadpu J., Singh A., Gupta K.R., Chandra N. (2014). Identifying feasible metabolic routes in *Mycobacterium smegmatis* and possible alterations under diverse nutrient conditions. BMC Microbiol..

[94] Bhaskar A., De Piano C., Gelman E., McKinney J.D., Dhar N. (2018). Elucidating the role of (p) ppGpp in mycobacterial persistence against antibiotics. IUBMB Life.

[95] Costerton J.W., Stewart P.S., Greenberg E.P. (1999). Bacterial Biofilms: A Common Cause of Persistent Infections. Science.

[96] Arora G., Sajid A., Virmani R., Singhal A., Kumar C.M.S., Dhasmana N., Khanna T., Maji A., Misra R., Molle V. (2017). Ser/Thr protein kinase PrkC-mediated regulation of GroEL is critical for biofilm formation in Bacillus anthracis. NPJ Biofilms Microbiomes.

[97] Siddam A.D., Zaslow S.J., Wang Y., Phillips K.S., Silverman M.D., Regan P.M., Amarasinghe J.J. (2021). Characterization of Biofilm Formation by *Mycobacterium chimaera* on Medical Device Materials. Front. Microbiol..

[98] Vega-Dominguez P., Peterson E., Pan M., Di Maio A., Singh S., Umapathy S., Saini D.K., Baliga N., Bhatt A. (2020). Biofilms of the non-tuberculous Mycobacterium chelonae form an extracellular matrix and display distinct expression patterns. Cell Surf..

[99] Katoch P., Gupta K., Yennamalli R.M., Vashistt J., Bisht G.S., Shrivastava R. (2019). Random insertion transposon mutagenesis of *Mycobacterium fortuitum* identified mutant defective in biofilm formation. Biochem. Biophys. Res. Commun..

[100] Falkinham J.O. (2018). *Mycobacterium avium* complex: Adherence as a way of life. AIMS Microbiol..

[101] Esteban J., García-Coca M. (2018). Mycobacterium Biofilms. Front. Microbiol..

[102] Dokic A., Peterson E., Arrieta-Ortiz M.L., Pan M., Di Maio A., Baliga N., Bhatt A. (2021). *Mycobacterium abscessus* biofilms produce an extracellular matrix and have a distinct mycolic acid profile. Cell Surf..

[103] Sharma S.K., Upadhyay V. (2020). Epidemiology, diagnosis & treatment of non-tuberculous mycobacterial diseases. Indian J. Med. Res..

[104] Ratnatunga C., Lutzky V.P., Kupz A., Doolan D.L., Reid D.W., Field M., Bell S., Thomson R.M., Miles J.J. (2020). The Rise of Non-Tuberculosis Mycobacterial Lung Disease. Front. Immunol..

[105] Faria S., Joao I., Jordao L. (2015). General Overview on Nontuberculous Mycobacteria, Biofilms, and Human Infection. J. Pathog..

[106] Sousa S., Bandeira M., Carvalho P.A., Duarte A., Jordao L. (2015). Nontuberculous mycobacteria pathogenesis and biofilm assembly. Int. J. Mycobacteriol..

[107] Victoria L., Gupta A., Gómez J.L., Robledo J. (2021). Mycobacterium abscessus complex: A Review of Recent Developments in an Emerging Pathogen. Front. Cell. Infect. Microbiol..

[108] Degiacomi G., Sammartino J.C., Chiarelli L.R., Riabova O., Makarov V., Pasca M.R. (2019). Mycobacterium abscessus, an Emerging and Worrisome Pathogen among Cystic Fibrosis Patients. Int. J. Mol. Sci..

[109] Ojha A., Hatfull G.F. (2007). The role of iron in *Mycobacterium smegmatis* biofilm formation: The exochelin siderophore is essential in limiting iron conditions for biofilm formation but not for planktonic growth. Mol. Microbiol..

[110] Ojha A., Anand M., Bhatt A., Kremer L., Jacobs W., Hatfull G.F. (2005). GroEL1: A Dedicated Chaperone Involved in Mycolic Acid Biosynthesis during Biofilm Formation in Mycobacteria. Cell.

[111] Chakraborty P., Bajeli S., Kaushal D., Radotra B.D., Kumar A. (2021). Biofilm formation in the lung contributes to virulence and drug tolerance of *Mycobacterium tuberculosis*. Nat. Commun..

[112] Chakraborty P., Kumar A. (2019). The extracellular matrix of mycobacterial biofilms: Could we shorten the treatment of mycobacterial infections?. Microb. Cell.

[113] Basaraba R.J., Ojha A.K. (2017). Mycobacterial Biofilms: Revisiting Tuberculosis Bacilli in Extracellular Necrotizing Lesions. Microbiol. Spectr..

[114] Taylor C.M., Beresford M., Epton H.A.S., Sigee D.C., Shama G., Andrew P.W., Roberts I.S. (2002). Listeria monocytogenes relA and hpt Mutants Are Impaired in Surface-Attached Growth and Virulence. J. Bacteriol..

[115] Lemos J., Brown T.A., Burne R.A. (2004). Effects of RelA on Key Virulence Properties of Planktonic and Biofilm Populations of *Streptococcus mutans*. Infect. Immun..

[116] De Paz L.E.C., Lemos J., Wickstrom C., Sedgley C.M. (2012). Role of (p) ppGpp in Biofilm Formation by *Enterococcus faecalis*. Appl. Environ. Microbiol..

[117] He H., Cooper J.N., Mishra A., Raskin D. (2012). Stringent Response Regulation of Biofilm Formation in *Vibrio cholerae*. J. Bacteriol..

[118] Schorey J.S., Sweet L. (2008). The mycobacterial glycopeptidolipids: Structure, function, and their role in pathogenesis. Glycobiology.

[119] Arora K., Whiteford D.C., Lau-Bonilla D., Davitt C.M., Dahl J.L. (2008). Inactivation of lsr2 results in a hypermotile phenotype in *Mycobacterium smegmatis*. J. Bacteriol..

[120] Pereira A.C., Ramos B., Reis A.C., Cunha M.V. (2020). Non-Tuberculous Mycobacteria: Molecular and Physiological Bases of Virulence and Adaptation to Ecological Niches. Microorganisms.

[121] Falkinham I.J. (2009). Surrounded by mycobacteria: Nontuberculous mycobacteria in the human environment. J. Appl. Microbiol..

[122] Wexselblatt E., Oppenheimer-Shaanan Y., Kaspy I., London N., Schueler-Furman O., Yavin E., Glaser G., Katzhendler J., Ben-Yehuda S. (2012). Relacin, a Novel Antibacterial Agent Targeting the Stringent Response. PLoS Pathog..

[123] De La Fuente-Núñez C., Reffuveille F., Haney E.F., Straus S., Hancock R. (2014). Broad-Spectrum Anti-biofilm Peptide That Targets a Cellular Stress Response. PLoS Pathog..

[124] Tkachenko A.G., Kashevarova N.M., Sidorov R.Y., Nesterova L.Y., Akhova A.V., Tsyganov I.V., Vaganov V.Y., Shipilovskikh S.A., Rubtsov A.E., Malkov A.V. (2021). A synthetic diterpene analogue inhibits mycobacterial persistence and biofilm formation by targeting (p) ppGpp synthetases. Cell Chem. Biol..

[125] Syal K., Flentie K., Bhardwaj N., Maiti K., Jayaraman N., Stallings C.L., Chatterji D. (2017). Synthetic (p)ppGpp Analogue Is an Inhibitor of Stringent Response in Mycobacteria. Antimicrob. Agents Chemother..

[126] Syal K., Bhardwaj N., Chatterji D. (2016). Vitamin C targets (p)ppGpp synthesis leading to stalling of long-term survival and biofilm formation in Mycobacterium smegmatis. FEMS Microbiol. Lett..

[127] Wang B., Dai P., Ding D., Del Rosario A., Grant R.A., Pentelute B.L., Laub M.T. (2018). Affinity-based capture and identification of protein effectors of the growth regulator ppGpp. Nat. Chem. Biol..

[128] Sun Z., Wu R., Zhao B., Zeinert R., Chien P., You M. (2021). Live-Cell Imaging of (p) ppGpp with RNA-based Fluorescent Sensors. bioRxiv.

